# Exploring pastoral attitudes and lived experiences of people living with HIV and/or AIDS: A case study of churches in Thulamela Municipality, South Africa

**DOI:** 10.4102/jphia.v17i1.1416

**Published:** 2026-04-03

**Authors:** Tshifhiwa S. Netshapapame, Cairo B. Ntimana

**Affiliations:** 1 Department of Public Health, Faculty of Health Care Sciences, University of Limpopo, Polokwane, South Africa; 2 DIMAMO Population Health Research Centre, Faculty of Health Care Sciences, University of Limpopo, Polokwane, South Africa

**Keywords:** HIV, AIDS, attitude, normalise, stigma, pastor, Christians, combat

## Abstract

**Background:**

The role of the church in addressing healthcare disparities, particularly human immunodeficiency virus (HIV) and/or acquired immunodeficiency syndrome (AIDS), and how pastors’ attitudes influence the development and effectiveness of HIV and/or AIDS interventions remains underexplored.

**Aim:**

This study aimed to examine pastors’ attitudes towards people living with HIV (PLWH) and to explore the lived experiences of PLWH in relation to these attitudes.

**Setting:**

Participants included pastors and congregational members living with HIV who resided in Thulamela Municipality and were affiliated with various Christian denominations.

**Methods:**

A qualitative phenomenological design was employed using semi-structured interviews. Purposive sampling was used to recruit nine pastors, while six PLWH were recruited through snowball sampling. All participants resided in Thulamela Municipality.

**Results:**

Findings indicated that pastors generally expressed sympathetic, non-judgemental and normalising attitudes towards PLWH. However, discussions around sexuality and HIV and/or AIDS were often considered taboo within the church context. People living with HIV reported experiencing stigma, and a prevailing belief among congregants attributed HIV transmission to individuals outside the church. Some participants also associated HIV infection with demonic possession.

**Conclusion:**

While pastors demonstrated compassion towards PLWH, inadequate knowledge contributed to persistent negative attitudes and stigma. Enhanced collaboration among pastors, interdenominational forums, healthcare professionals and communities is essential to promote accurate information sharing, reduce stigma and improve HIV and/or AIDS responses within faith-based settings.

**Contribution:**

This study highlights how pastor-led biblical discourse, influenced by limited theological training, shapes attitudes towards HIV and/or AIDS and underscores the need for stronger collaboration between churches and public health.

## Introduction

The role of the churches as an agent of change is one of the significant discussions in public health and communities worldwide.^1,2,3^ The same role that the church plays has been documented in Africa.^4,5,6^ For instance, in South Africa, churches have been identified as instrumental in healthcare delivery, especially where health sectors are underdeveloped.^7,8^ They are involved in various community health organisations, and they contribute significantly to public health, although their role is often overlooked.^7,8^ Within rural communities, the churches uphold and reinforce societal and religious core values and beliefs such as justice, morals, compassion, faith and spirituality.^9,10^ Moreover, they serve as a source of stability and solace for the community by engaging in outreach, political activism and promoting healthcare initiatives.^11^ As a result, African pastors can play a pivotal role as stakeholders in the battle against human immunodeficiency virus (HIV) and/or acquired immunodeficiency syndrome (AIDS) by disseminating education and crucial information to their congregants and communities regarding prevention, testing and treatment options for HIV and/or AIDS.^3^

Although the church has played a good role in public health, not all pastors and church communities are welcoming of marginalised people. In some cases, organised religion creates labels and stereotypes that stigmatise and vilify many marginalised groups.^4,12,13^ For example, some faith-based organisations are found to be in denial regarding HIV and/or AIDS,^14,15,16^ with pastors not perceiving HIV and/or AIDS to be a risk to their communities.^17^ Despite the involvement of pastors and Christian-based organisations in combating the spread of HIV and new infections, stigma and discrimination persist as significant barriers to HIV and/or AIDS prevention and care initiatives within church settings.

Netshapapame et al.^18^ allude to the historical role of the church as a social institution deeply embedded in its context, often providing sanctuary and support to marginalised individuals. For instance, mission stations offered refuge to young girls fleeing forced marriages, and missionaries extended hospitality to those marginalised by cultural norms and practices. In the South African context, the church played a pivotal role during apartheid, offering shelter to the destitute and actively supporting the anti-oppression movement.^17^ However, it is important to acknowledge that the church has not always upheld these ideals. Emerging literature highlights instances where the church has also been implicated in harmful practices, including gender-based violence (GBV), financial mismanagement and abuse of power, which have compromised its role as a safe space for vulnerable populations.^19,20^ Thus, while the church has historically contributed positively to social justice, a more balanced understanding must also consider the contradictions and moral failings present within some religious institutions.^21^ It is on that note that theologians have shown that the church today must be cognisant of the fact that HIV and/or AIDS is real, it does exist within the church, and the church cannot turn a blind eye to HIV and/or AIDS.^22^

Another challenge arises from the close connection between HIV and AIDS, sexuality and shame.

Human immunodeficiency virus-related stigma can stem from a variety of factors, including misinformation, cultural norms, social judgement and religious beliefs. In some contexts, stigma is reinforced by religious interpretations that view HIV as a moral failing or divine punishment, which may lead to shame and marginalisation of people living with HIV and/or AIDS (PLWHA).^23^ In addition, the stigma and shame associated with HIV and/or AIDS may influence the reluctance of pastors to engage in open discussions about HIV and/or AIDS. This hesitation further reinforces the stigma surrounding HIV and/or AIDS.^24,25^ Furthermore, it has been noted that many pastors in Southern Africa did not explicitly criticise HIV and/or AIDS management strategies. While certain religious beliefs and a perception of spiritual purity have, at times, hindered open discussions about sex and HIV transmission, topics often considered taboo within church settings.^18,26,27^ Many Pentecostal and Catholic churches across Africa have actively supported PLWHA, offering counselling services, providing space for support groups, facilitating HIV counselling and testing and initiating community-based projects aimed at improving the lives of PLWHA.^4^

In Mozambique, it was reported that pastors faced challenges in addressing HIV and/or AIDS-related stigma because of a lack of appropriate vocabulary and confidence.^28^ Moreover, pastors were hesitant to attempt such discussions because of ongoing opposition from senior pastors.^28^ The integration of HIV and/or AIDS into existing religious frameworks reinforces stigma, making it difficult to discuss the issue without invoking concepts of immorality and shame.^27^ This limitation restricts the potential for critical examination of stigma, which is crucial for developing more constructive and less judgemental perspectives and responses to HIV and/or AIDS.^29^

Researchers are essentially in agreement that pastors have been part of the problem rather than being part of the solution.^30^ Pastors have faced criticism for their delayed responses, their reluctance to acknowledge the magnitude and significance of increasing HIV and AIDS infection rates and for perpetuating silence and secrecy surrounding the issue.^30^ The association of HIV with certain behaviours, such as premarital sex, extramarital affairs or drug use, and the hesitation to openly address these sensitive issues within church environments have contributed to the stigma and discrimination experienced by individuals living with HIV.^29,31^ Examining the role of the church in addressing healthcare disparities, particularly about HIV and/or AIDS, and understanding how pastors’ attitudes towards people living with HIV (PLWH) affect the development and effectiveness of HIV and/or AIDS programmes within communities remains largely unexplored. Given the role pastors play in communities, their attitude may affect the health-seeking behaviour of PLWH. Simultaneously exploring the attitude of pastors with the lived experiences of PLWH may assist in creating a holistic support system for PLWH. Thus, the primary aim of the study was to explore the attitudes that pastors have towards PLWH. The study also aimed to investigate the lived experiences of PLWH within the church settings.

## Research methods and design

### Study design

A qualitative phenomenological design was adopted.^32,33,34^ This study design employs a flexible methodology that generates high-quality data, facilitating a deeper comprehension of the attitudes pastors hold towards individuals living with HIV or AIDS and the lived experiences of people with HIV or AIDS.^32,33,34^

### Study site

The study was conducted in the Thulamela Municipality of Limpopo province, South Africa. The study area is 5834.70 km^2^ with a population of approximately 618 462 (106.00 per km^2^), and 156 594 households (26.84 per km^2^). As of 2022, Limpopo recorded an HIV prevalence rate of 8.9%, a decrease from 10.1% in 2017, which translates to approximately 570 000 PLWH in the province.

### Sampling and study population

The sample consisted of pastors and PLWH from various denominations, including African churches (Zion), Lutheran, Presbyterian, Roman Catholic, Methodist and Charismatic churches within the Thulamela Municipality. Purposive sampling was employed to select pastors, while snowball sampling was used to recruit PLWH. The study included lead or head pastors of congregations and members of those congregations who are living with HIV. Congregational members who were not living with HIV and were neither leaders nor lead pastors were excluded from the study.

Participants aged 50 years and above were deliberately selected. This decision was based on the study’s aim to engage with individuals who have extensive experience and historical insight into the church’s evolving response to HIV and/or AIDS. Senior pastors were deemed more likely to have ministered during critical periods of the epidemic, while older PLWH were able to provide retrospective accounts of their interactions with church support systems. In total, the study included 15 individuals, eight pastors, one bishop and six congregational members from nine different churches.

### Data collection

In-depth face-to-face interviews were conducted with both pastors and PLWH who consented to be part of the study. The research team explained the purpose and objectives of the study to lead pastors and PLWH. Pastors and PLWH were assured of the voluntary nature of their participation and were informed of their right to withdraw from the study at any point. None of the participants withdrew from the study. Interview appointments were scheduled with each participant at a location they deemed safe, private and free from disturbances. These locations included homes and church halls. Interviews were conducted in private rooms by T.S.N. (male, PhD), R.G.M. (male, BSc), C.B.N. (male, MSc), K.P.S. (male, MSc) and E.M. (male, PhD), who are experienced qualitative researchers. The interviews were conducted using a focused group discussion that consisted of socio-demographic characteristics and open-ended questions. The researchers used audio recorders along with notepads to record the interviews, which lasted approximately 45 min. Data were collected until data saturation was reached at participant number 13 (both pastors and PLWH). Only the research team and participants were present during the data collection.

### Data analysis

Qualitative content analysis was used as recommended by Creswell and Guetterman^35^ to analyse the data. A team of two researchers organised the data, including transcribing all recorded audio and field notes. They read the transcripts to gain a general understanding of the material. The next step involved coding the data, which included identifying segments to assign code labels. These codes were then analysed to develop themes and their descriptions. A meeting with additional authors was held to identify and refine these themes. The themes were then reported as qualitative findings. Ultimately, all authors agreed on the themes and sub-themes presented in Table 1. By using content analysis, the research team was able to interpret the text data through a systematic coding classification process. Through intense immersion in the data, the team allowed new insights to emerge, resulting in the themes and sub-themes outlined in Table 1.

**TABLE 1:** Summary of the results.

Themes	Sub-themes
1. Positive attitudes towards people living with HIV	1.1. Non-judgemental attitude 1.2. Normalising HIV discussions and disclosure 1.3. Sympathising with PLWH
2. Negative attitudes towards people living with HIV	2.1. Addressing topics related to sexuality and HIV and/or AIDS within the context of the church is often perceived as taboo 2.2. Language barriers 2.3. Cultural barriers 2.4. Imbalance in preaching about holiness that daily issues affecting congregants 2.5. Stigmatisation 2.6. Blaming the non-church members as the ones who infect Christians 2.7. Relating HIV infection as a result of possessions by demons

HIV; human immunodeficiency virus; AIDS, acquired immunodeficiency syndrome; PLWH, people living with HIV.

### Measures to ensure trustworthiness

To ensure the accuracy and rigour of the study, the researchers followed Lincoln and Guba’s four evaluation criteria, which offer a structured framework for assessing the trustworthiness of qualitative research.^35^ Credibility was established through a member-checking process involving feedback from three participants and the authors, ensuring the accuracy of the interpretations. Adequate time was allocated to data collection, analysis and engagement with the data, improving the study’s validity. Transferability was addressed by providing detailed descriptions of the research setting, participant demographics and methods, allowing readers to evaluate the applicability of the findings to other contexts. Including participants with diverse characteristics and using rich, illustrative quotations further enhanced the contextual relevance of the findings. To ensure confirmability, the researchers maintained an audit trail documenting the study’s procedures, decisions and adjustments, enabling verification of the findings. Consistency was improved by standardising the interview process, ensuring that all participants answered the same set of questions. Dependability was achieved through transparent documentation of all research activities and rigorous review by additional researchers, providing an external check on the accuracy and reliability of the process.

### Ethical considerations

Ethical clearance to conduct this study was obtained from the University of South Africa’s Health Studies Higher Degrees Ethics Review Committee (No. HSHDC/976/2020).

## Results

Table 2 illustrates the demographic characteristics of participants, with the majority being male and over the age of 50 years.

**TABLE 2:** Demographic data of the participants.

Pseudonyms	Age (years)	Gender	Type denomination
Pastor 1	56	Male	Charismatic
Pastor 2	54	Male	Charismatic
Pastor 9 (Bishop)	60	Male	Charismatic
Pastor 3	65	Male	Charismatic/Main Line
Pastor 4	60	Male	Charismatic
Pastor 5	67	Male	Charismatic
Pastor 6	62	Female	Charismatic
Pastor 7	53	Male	Charismatic
Pastor 8	54	Male	Charismatic
Participant 1	56	Female	Charismatic
Participant 2	58	Female	Charismatic
Participant 3	68	Male	Zion
Participant 4	64	Male	Charismatic
Participant 5	50	Male	Charismatic
Participant 6	70	Male	Zion

### Themes

Two overarching themes of pastors’ attitudes towards HIV and AIDS emerged, which encompassed two distinct themes: positive and negative attitudes, both of which have significant implications for the lives of PLWH. The sub-themes for the positive attitude themes revealed the attitude of non-judgement, normalised attitudes and sympathetic attitudes towards PLWH. Perceptions of the topic of HIV being taboo, stigmatisation and blame and relating HIV to demon possessions have been noted as sub-themes to the negative attitude theme (Table 1).

#### Theme 1: Positive attitudes towards people living with HIV

This theme focuses on pastors’ attitudes that positively influence the well-being of PLWH. The analysis revealed the emergence of the following sub-themes: non-judgemental attitude, normalisation and empathy towards those who are infected.

**Sub-theme 1.1: Non-judgemental attitude:** Pastors have emphasised showing compassion and unconditional love to PLWH, for they regard PLWH as normal as other church members with or without other conditions. Pastors offer reassurance that HIV is a manageable health condition and emphasise the importance of continued medication adherence. The pastors further mentioned that the act of non-judgement and compassion towards PLWH was a biblical standpoint of loving one’s neighbour as oneself. Thus, individuals living with HIV or AIDS are considered neighbours, deserving of love, non-judgement and compassion. The pastors further highlighted that living with HIV or AIDS does not negate one’s status as a neighbour; rather, it emphasises the importance of the church’s obligation to love and support them. Additionally, the pastors stated that they view PLWH as their children and support them just as they would for individuals with any other health condition. The pastors were also found to encourage PLWH to adhere to their medicine as they (pastors) continued to offer them prayers. The following assertions illustrate these findings:

‘I take PLWH as my children like others. A person with HIV I takes that like any person who has got a chronic disease. To me, HIV is like any other disease. It is not all HIV-positive people who happen to be living a reckless life like what most people perceive. It does not make any sense that I have to find myself being uncomfortable while I am with a person with HIV while I am comfortable with a person with Sugar Diabetes or hypertension, so to me, I handled them the same.’ (Pastor 9, 60 years, male)

Similar perceptions were shared by PLWH. People living with HIV noted that they experienced less negativity from their pastors and felt encouraged to take their medication. The following extracts illustrate these findings:

‘In our church, my pastor is the person who loves all people despite their background, so he does not condone stigmatization in the church. At church, I sing, and I am a member of the worship team, so the day I disclosed this to him he said you need to continue doing what you do best without any hindrance.’ (Participant 1, 56 years, female)

Another person living with HIV highlighted that congregants are still discriminating against PLWH. This was the case during conferences or other organised events, where PLWHs were isolated and relegated for fear of infection. The participant had this to say as PLWH in the church:

‘During the conference, we are being given a job to make fire, we are not allowed to peel vegetables, but we are given this lousy job and are not involved in critical work because people are afraid that we might infect the food.’ (Participant 3, 68 years, male)

This finding reveals that even though pastors are striving to be non-judgemental and accommodating to PLWH, some congregants discriminate against PLWH.

**Sub-theme 1.2: Normalising HIV discussions and disclosure:** This study’s findings revealed that pastors portray normalised attitudes towards PLWH and normalise discussions about HIV and/or AIDS within the church. This is the case when congregants disclose their HIV status. Pastors reported that they try to react normally after realising that members are HIV-positive. They further indicated that they treat HIV as any other chronic condition. The following excerpts attest to the response of a pastor’s normalised attitude:

‘Congregants would come and say, pastor, I am HIV-positive. I would not show any expression as if I am shocked, for as a parent, I need to react normally for the benefit of the PLWH. Thereafter, I tell the PLWH that there is life after contracting HIV, and we start to look for a way forward because we cannot change the past.’ (Pastor 4, 64 years, male)

The study further reveals that pastors have played a pivotal role in fostering an inclusive and supportive church environment. By promoting acceptance and compassion, they have helped reduce fear and judgement, allowing PLWH to openly share their status and feel fully integrated into the church community, treated with the same dignity and respect as any other member. The following quotation supports these findings:

‘The pastor has made it a point that HIV is being taken like any sickness. When I give announcements, I always talk about HIV and encourage people to always be alert about HIV. Talking about HIV/AIDS in our church is easy for the pastor has created a conducive atmosphere.’ (Participant 5, 50 years, male)

**Sub-theme 1.3: Sympathising with the infected person:** This study revealed that pastors sympathise with PLWH, giving them physical, emotional, psychological and spiritual support.

Participants further emphasised the importance of PLWH feeling accepted and supported by their loved ones and the broader community, as this encouragement helps them adhere to their treatment and maintain hope for the future. Pastors show sympathy in assuming the role of reconciling and mediatory between the destitute PLWH and their families. People living with HIV and their families have been receiving pastoral care and counselling from pastors:

‘I have sympathy for those who have been impacted by HIV/AIDS. I always sympathize with them because, in most cases, they are being rejected by their families. I go and offer a word of encouragement, reconcile PLWH with the family.’ (Pastor 2, 54 years, male)

‘Sometimes you find families at loggerheads with each other because parents will be saying we used to rebuke the infected but could not listen so now is payback time for the ill-behaviour. So as a pastor, I come in between to forge the broken relationships and seek forgiveness on behalf of the PLWH to the family.’ (Pastor 9, 60 years, male)

#### Theme 2: Negative attitudes towards people living with HIV

Alongside the positive attitudes that contribute to the well-being of PLWH, this theme highlights that pastors have also displayed negative attitudes that can have adverse effects on PLWH. The following sub-themes emerged from data analysed from the interviews: regarding discussions about sexuality and HIV and/or AIDS in church as taboo, stigmatisation of PLWH, attributing blame to non-church members for infecting Christians, and associating HIV infection with demonic possession (Table 1).

**Sub-theme 2.1: Addressing topics related to sexuality and HIV and/or AIDS within the context of the church is often perceived as taboo:** The present study revealed that addressing topics related to sexuality and HIV and/or AIDS within the context of the church is often perceived as a religious taboo. This taboo poses a significant obstacle to HIV prevention efforts and sexual and reproductive health services for PLWH. The present study further highlighted that cultural barriers remain a stumbling block for HIV through the limiting of free talk about HIV. For instance, it has been revealed that in the Tshivenda traditions, from which the churches were located, sexuality matters are not supposed to be articulated with young people in attendance. It is not only problematic but also chaotic. The following extracts illustrate these findings:

‘This is true I will talk from the experience where one of my associate pastors made mention of sexual intercourse while preaching, and that has created serious chaos inside the church. You must remember that our church has got a lot of older people. A complaint was lodged with me to say that, that was not supposed to be said moreover we had children in attendance. In our culture, it is taboo to talk about sexual intercourse.’ (Pastor 9, 60 years, male).

**Sub-theme 2.2: Language barriers:** Apart from the perception that topics related to sexuality and HIV and/or AIDS within the context of the church are taboo, the present study found that there were some language barriers making it difficult to address topics related to sexuality and HIV and/or AIDS. It was expressed that some Tshivenda words are difficult to utter in public:

‘Culture is also contributing to hindering us as pastors to speak out on HIV and AIDS. There are some words and terms in our Tshivenda language that we as a Christian community are tough to utter in public e.g., sexual intercourse.’ (Pastor 3, 65 years, male)

**Sub-theme 2.3: Cultural barriers:** Within African churches, matters related to sexuality are often viewed as private, and individuals who broach these topics are perceived as inexperienced and disrespectful. The results of the current study illustrate that the church, even though it is strategically situated in the community to address issues that concern HIV or AIDS, is unable to fulfil its duties because of the cultural barriers and the language gap. Participants expressed the need for the church to dismantle cultural barriers and foster open, fear-free discussions about HIV within church settings. They emphasised that such openness is essential for reducing stigma and promoting awareness and support for PLWH.

The participants argued that people would die of HIV and/or AIDS while the church remained silent at such a time when the church, as one of the critical stakeholders, was supposed to be vocal. This attitude affects PLWH and the entire community to benefit from their Sunday church services over and above the day’s sermon. Most participants living with HIV expressed scepticism towards the church’s focus on preaching holiness and righteousness while failing to address the realities of HIV. They felt that this silence contributed to unnecessary suffering and believed the church should have taken a more active role in educating members about HIV transmission and prevention. As one participant stated:

‘So, it is incumbent upon the church to start to educate its members about the impact that HIV has got in a family setup. This health promotion and education on HIV need to start with Sunday school kids. Thus, in the day sermon pastor must dedicate a few minutes to talk about health-related issues beyond HIV/AIDS.’ (Participant 5, 50 years, male)

**Sub-theme 2.4: Imbalance in preaching about holiness that daily issues affecting congregants:** This study revealed that churches are preaching more on holiness and turn to relegating the issues that affect people daily, which include HIV and AIDS and other health-related matters. The participants voiced their concerns that it is time that the church be brave enough to stand beyond the cultural barriers and start to talk about sexuality as early as with Sunday school kids. The following quotation supports these findings:

‘The challenge that I have to pick up is that at church, we speak of holiness, and we tend not to delve deep into issues that affect people like HIV/AIDS.’ (Participant 1, 56 years, female)

**Sub-theme 2.5: Stigmatisation:** These findings reveal that stigmatisation is still rife in the church for their pastors who still believe that HIV is transmitted through adulterous activities. Limited information and awareness, coupled with outdated beliefs, contribute to a fear of contracting HIV. The above findings concur with the sentiments shared below:

‘The reason is that most Christians failed to understand that HIV AIDS is like any other sickness. They are afraid to come out because they are afraid of being stigmatized that they will be referred to as people who are adulterous and careless. At the same time, there are so many ways that people can be infected by HIV and AIDS other than sexual intercourse.’ (Participant 2, 58 years, female)

Over and above stigmatisation, PLWHs are being discriminated against at church by being given less critical work in fear of HIV infection:

‘Sometimes church members are the problematic ones, not pastors when it comes to acknowledging people living with HIV and AIDS. When there are conferences, PLWHs are given a lousy job compared to preparing food for fear that they might infect the food.’ (Participant 1, 56 years, female)

The findings of this study indicate that within many religious circles, HIV is still perceived as a death sentence and is often linked to moral failings, particularly adultery. Such perceptions have contributed to fear, stigma and discrimination within church communities. Participants reported that some congregants avoid eating food prepared by PLWH because of irrational fears of transmission. This stigmatisation is deeply rooted in earlier church teachings that associated HIV and/or AIDS with sin and divine punishment. Notably, pastors were identified as key figures in reinforcing these harmful narratives, both through their preaching and attitudes, which have discouraged open dialogue and support for PLWH within the church.

The following quotes illustrate these sentiments:

‘When this sickness started, I was among people who criticized HIV-positive people … this sickness was associated with adulterous behaviour and being careless. My experience with this sickness is very painful because I used to stand up behind the pulpit and criticize PLWH. This in a way caused a lot of pain to people who have been victims of these HIV/AIDS.’ (Pastor 9, 60 years, male)

‘However, many people are still afraid to speak out of their status since a lot of people are still associating HIV and AIDS with the sin of adultery.’ (Pastor 2, 54 years, male)

‘HIV has been associated with ill-behaviour discouraging people to come to us pastors with fear to be judged … It is easier for PLWH to confide to people outside the church than with the pastor for the fear of being judged.’ (Pastor 6, 62 years, female)

‘There are pastors who regard us PLWH as people who had an adulterous history … At times it is difficult even to secure an appointment with the pastor for most of them do not want to associate with us for fear of HIV.’ (Participant 1, 56 years, male)

**Sub-theme 2.6: Blaming the non-church members as the ones who infect the Christians:** Pastors claim that as Christians live a holy life, the virus has been brought to them by their unbeliever partners who were living promiscuous lives. This study reveals that pastors blame non-church members for the ones infecting Christian members. This is supported by the narrative:

‘… Ok! let me start by saying when this sickness started, I was among people who criticized people who were HIV-positive. Because always this sickness was associated with adulterous behaviour and being careless. As time goes on when we started to have the people inside the church who were infected and affected by HIV AIDS, People whom you know very well that they have been living faithful life. So, when you start to get involved you realise that the woman was living a faithful life but this infection was brought back home by the husband who was leaving unfaithful life. What hit me so hard was one person who was closer to me and my wife. She came and confide it with me saying that ever since she was born, she never had any sexual intercourse with anybody other than her husband the most worrying factor was that the husband kept on blaming the wife that she’s the one who is the cause of the sickness. My experience with this sickness is very much painful for the reason that I used to stand up behind pulpit and criticize people living with HIV.’ (Pastor 9, 60 years, male).

‘… Yes, while I was still working for the Department, I found out I was HIV-positive. I was very worried and anxious that my husband who is not into church stuff infected me … I went to counselling. I was told that there is no need to cry over spilt milk and I should try to look forward to the future. I was counselled and told that I need not to divorced because I was contemplating to lodge a divorce … I was told to sit down with my partner and talk through this and live a healthy life for there is still life after being infected by HIV.’ (Participant 2, 58 years, female)

**Sub-theme 2.7: Relating HIV infection to possession by demons:** The participants noted that HIV and/or AIDS is perceived as a manifestation of demonic influence, and individuals affected by the illness are seen as targets of spiritual attacks. These deliverance rituals negatively affect the health of PLWH as they diminish the need to take treatment. This can be alluded to in the following quotations:

‘They even go up to the extent of saying that they had incidents where people were said to be having demons but later found that those people got tested and they were found to be HIV-positive.’ (Pastor 7, 53 years, male)

‘Biblically, they might be partly wrong and partly right because the life we are living starts in the spirit and then manifest in the physical. However, HIV and AIDS are more physical than spiritual and are being transmitted physically through sexual intercourse and contaminated blood contact. HIV is a spirit, you cannot reject it, but the truth is it is more physical than.’ (Pastor 5, 67 years, male)

Besides the attitudes that the pastors have explicated, there are practices that pastors are explicating towards HIV and/or AIDS in churches. The participants revealed that PLWH are vulnerable and exposed to false prophets who discourage them from adhering to their medication, thereby compromising their health and treatment outcomes. The participant also indicated that false prophets are propelling messages that undermine the effect of normalising HIV and/or AIDS:

‘These false prophets and pastors are the ones who are always propelling this propaganda that there is a different kind of HIV that is demonic, they are sowing a bad seed in the minds of people who are infected by HIV and that is irresponsible.’ (Participant 1, 56 years, male)

## Discussion

The study aimed to explore the pastors’ attitudes towards PLWH and to investigate the lived experiences of PLWH. The findings of the present study revealed that the pastors have not been judgemental towards PLWH; instead, they have shown love and compassion. Moreover, the PLWH indicated that they are happy at church and that both preaching and personal support from pastors encourage them to adhere to their HIV medication. This finding aligns with the literature from UNAIDS,^36^ which has reported that pastors need to show compassion and love to PLWH and further support them in their endeavours in the church. However, alongside these positive attitudes that contribute to the well-being of PLWH, this theme also highlights that some pastors have displayed negative attitudes that could have adverse effects on PLWH. These findings imply that HIV prevention and control programmes could benefit from integrating faith-based components that promote compassion-driven counselling and adherence support within church settings.

Previous studies indicated that pastors are willing to engage in open, honest conversations about HIV and are supportive of PLWH,^37,38,39,40^ which aligns with the findings of the present study. However, some participants shared contrasting experiences, noting that certain congregants still display discriminatory attitudes towards PLWH. They noted that during conferences or events, PLWH are isolated and assigned routine tasks because of fears of infection. This finding reveals that even though pastors are striving to be non-judgemental and accommodating to PLWH, more work still needs to be done to educate congregants about HIV and/or AIDS to eliminate stigmatisation and discrimination of PLWH and normalise attitudes towards PLWH.^41,42^ Therefore, HIV prevention and control initiatives should include stigma-reduction training and sensitisation workshops for both pastors and congregants to strengthen community-level acceptance and reduce discrimination within faith communities.

In addition to normalising talking about HIV and/or AIDS within the church, pastors are demonstrating empathy towards individuals who are infected. The findings of the present study are in agreement with a study by Sivelä, who indicated that pastors are striving to normalise HIV in the church so that it must not be taboo to talk about it.^43^ In contrast to community-based studies such as those from India, research conducted within church settings has often highlighted the persistent stigma and discrimination faced by PLWH.^44,45^ Several studies have reported that some religious leaders and congregants continue to associate HIV with immoral behaviour, often linking infection to sin, promiscuity or divine punishment.^46,47^ However, other studies also indicate that with proper sensitisation and HIV education, churches can become powerful agents of change by promoting compassion, inclusion and care.^48,49^ This underscores the potential role of religious institutions as key partners in HIV prevention campaigns, particularly in delivering messages that challenge stigma and encourage early testing, treatment adherence and community inclusion.

Religious leaders ought to exhibit sensitivity towards the needs of PLWH and take on a significant role in fostering a culture of acceptance and respect towards them. This includes promoting ideas of responsibility and tolerance within their communities.^37,50^ Similar studies have found that pastors have established a secure and encouraging atmosphere that fosters community acceptance, emotional healing and open dialogue about HIV and AIDS among congregants.^47^ Pastors have also acted as intermediaries between PLWH and their families and communities, offering them spiritual, social and material support (food parcels). This study aligns with existing literature, indicating that pastors exhibit sympathy and compassion towards PLWH, encouraging them to maintain hope and envision a brighter future.^51,52,53,54^ Hence, faith-based organisations could serve as effective entry points for community-based HIV prevention and support programmes that address both the spiritual and psychosocial needs of PLWH.

However, while these positive attitudes support the well-being of PLWH, pastors have also shown negative attitudes that can cause harm to PLWH. The present study indicated that talking about sexuality and HIV and/or AIDS at Church is taboo; the pastors indicated that the cultural barrier remains a stumbling block for HIV through the limiting of free talk about HIV, illustrating that certain cultures do not condone talking about sexuality. In agreement with the present study, several studies reported similar findings.^55,56,57^ Similarly, the National Council of Churches in the Philippines conducted a study revealing a religious taboo surrounding discussions about sexuality, gender and sexual matters.^58^

The results of the current study illustrate that the church, even though it is strategically situated in the community to address issues that concern HIV and/or AIDS, is unable to fulfil its duties because of the cultural barriers and the language gap. The finding further indicated a need for the church to break down cultural barriers and begin openly discussing HIV within the church environment without fear. Creating safe spaces for dialogue and testimony may also empower PLWH to share their experiences and help reduce stigma within the faith community. Human immunodeficiency virus and/or AIDS control programmes should therefore prioritise partnerships with churches to establish such safe spaces and integrate faith-based education modules that encourage dialogue, disclosure and collective action against stigma.

The participants argued that people would die of HIV or AIDS while the church remained silent at such a time when the church, as one of the critical stakeholders, was supposed to be vocal.

The findings are consistent with a prior study conducted by Murungi,^59^ indicating that the church’s efforts in combating HIV and AIDS are still inadequate, given the challenges in addressing HIV, AIDS and sexuality. Most PLWH participants were sceptical about the church preaching about holiness and righteousness while members were dying in silence when the church could have done an enormous work of preaching about HIV and ways transmitted among church members and to the extent of talking about how it can be prevented. These findings are congruent with Dube’s^60^ findings that the church must exhibit courage and engage in discussions about HIV and/or AIDS.

This study’s findings revealed that some of the pastors continue to uphold the belief that HIV is the ultimate consequence of leading a sinful life, and they view it as a divine punishment inflicted by God. Sarur and Kaba^61^ indicated that pastors emphasised the belief that HIV and/or AIDS is a punishment from God for individuals who do not adhere to his teachings regarding abstinence before marriage and faithfulness thereafter. They attributed the spread of HIV and/or AIDS to various factors, including acts of adultery.^61^ This study’s findings concur with the previous study, as participants believe that HIV and/or AIDS is associated with adulterous conduct. Participants have revealed the fact that HIV is for sinners, not for Christians. Pastors always blame HIV on non-church members infecting Christians. These misconceptions highlight the urgent need for collaboration between public health authorities and faith leaders to dispel myths and integrate theological perspectives that align with scientific understanding, ultimately strengthening the impact of HIV and AIDS prevention and control programmes.

This study has several limitations that should be acknowledged. Firstly, the sample was predominantly composed of pastors and participants from charismatic denominations, with limited representation from Mainline and Pentecostal churches. While the findings offer valuable insights into the experiences of the participants, they may not be directly transferable to all Christian denominations or context. Efforts to include participants from other denominations were met with logistical constraints and limited willingness to participate. Secondly, all interviewed pastors were above the age of 50 years. This age-based selection was intentional, as the study aimed to gather insights from experienced clergy who have been involved in pastoral care over an extended period, particularly during the peak of the HIV and AIDS epidemic. However, this criterion may have excluded the perspectives of younger clergy who could offer contemporary or alternative approaches to pastoral support. Thirdly, this study did not explore pastors’ own HIV status, their relationships with PLWH or their sources of HIV-related knowledge. It also did not assess the presence or absence of church-based HIV and/or AIDS programmes. These factors are critical in shaping attitudes and responses towards PLWH. Their omission limits the depth of interpretation and practical recommendations. Future research should consider including a more diverse sample in terms of denomination, gender and age to provide a more comprehensive understanding of pastoral care practices.

## Conclusion

The study revealed that there is a good relationship between PLWH and pastors. Despite pastors showing love and compassion towards PLWH, some pastors still feel that HIV is associated with the infected person’s adulterous behaviour. People living with HIV still experience discrimination and stigmatisation from the church. The study revealed that PLWH often face subtle but hurtful forms of stigma within church settings. For instance, participants shared that during church conferences, they were assigned menial tasks such as making fires, while being excluded from food preparation because of unfounded fears that they might transmit the virus through handling food. Such actions reflect persistent misconceptions about HIV transmission within some congregations. Additionally, a belief persists that HIV is brought into the church by outsiders or non-members, creating a false narrative that isolates and blames others. This perception fosters a divisive environment and may prevent open dialogue and effective prevention strategies. The study also found that HIV and/or AIDS is still frequently associated with spiritual causes, with some church members viewing it as a manifestation of demonic possession or satanic attack. As a result, affected individuals often resort to religious rituals aimed at spiritual cleansing rather than seeking biomedical treatment or psychosocial support. These findings underscore the need for faith-based HIV education grounded in both theology and science to challenge stigma and misinformation. These rituals negatively affect the health-seeking behaviour, which makes them reluctant to take treatment. The church still has to win its battle with the cultural barriers that exist, hindering the church from addressing HIV and/or AIDS accordingly.

### Recommendations

The church, as the agent, needs to further stop discourse preaching that says HIV is a demon and needs spiritual warfare that includes fasting, leading the PLWH to default on their medication, thus compromising their health in the process. There is an urgent need for the collaboration of different stakeholders, which include pastors, pastors’ forums, interdenominational conferences, health practitioners and the community at large to curb discrimination and stigmatisation of PLWHA.
